# Psychiatric nurses’ experiences of using a model to improve relationships of couples with borderline personality disorder

**DOI:** 10.4102/sajpsychiatry.v31i0.2450

**Published:** 2025-04-15

**Authors:** Andile G. Mokoena-de Beer, Annie Temane, Nompumelelo Ntshingila

**Affiliations:** 1 Department of Nursing Science, School of Health Care Sciences, Sefako Makgatho Health Sciences University, Pretoria, South Africa; 2 Department of Nursing, Faculty of Health Sciences, University of Johannesburg, Johannesburg, South Africa

**Keywords:** borderline personality disorder, experiences, implementation, model, psychiatric nurses, relationships

## Abstract

**Background:**

Individuals with borderline personality disorder (BPD) struggle with relationships because of impulsivity and emotional regulation difficulties. Specialised skills are needed for care, but existing models are lacking. A new model was developed to help psychiatric nurses support couples with one partner having BPD. This innovative psychiatric nursing model emphasises the need for implementation and evaluation in care.

**Aim:**

To explore the psychiatric nurses’ experiences of implementation of a model to facilitate constructive intra- and interpersonal relationships for couples in a relationship where one is living with BPD.

**Setting:**

The study was conducted at a mental health institution in Gauteng province where the one partner with BPD receives treatment.

**Methods:**

The study adopted a qualitative, exploratory design conducting in-depth interviews with psychiatric nurses who implemented the model, triangulated with observations and field notes and analysed thematically.

**Results:**

Four themes emerged, namely: (1) the model served as a helpful tool for empowerment; (2) the positive results yielded by the model; (3) challenges related to using the model; and (4) suggestions for expanding the model’s reach. The psychiatric nurses found the model acceptable and feasible to facilitate the relationships of couples where one is living with BPD.

**Conclusion:**

Psychiatric nurses need guidelines to enhance care for individuals with BPD, highlighting the importance of implementing supportive models to improve relationships in various settings.

**Contribution:**

Implementation of this model is unique in the care of persons living with BPD making it a valuable tool for psychiatric nurses in mental health care provision.

## Introduction

### Background

Individuals with borderline personality disorder (BPD) are often not well accepted, specifically by psychiatric nurses and other professionals because of their behaviours,^1^ which include impulsivity, trouble regulating their emotions, mood changes including anger and unstable relationships linked to their inability to form strong bonds.^2^ This inability of individuals with BPD to form strong bonds affects therapeutic relationships with the psychiatric nurses also.^3^ Thus, caring for persons with BPD requires specific skills to improve relationships of those with BPD and their spouses.^4^ This can be achieved through education, training and support for the psychiatric nurses.^5^ However, O’Connell and Dowling^6^ noted in their study that training relating to caring for persons with BPD is insufficient. Therefore, the researchers of this study developed a model for psychiatric nurses to facilitate constructive intra- and interpersonal relationships for couples living with BPD. In this study, only one partner in the relationship had BPD. However, the model was applied to both as BPD affects the relationship with the self as well as with others. This article explores the psychiatric nurses’ experiences of implementing a model to facilitate constructive intra- and interpersonal relationships for couples in a relationship where one is living with BPD.

Models in psychiatric nursing practice can be used to guide the practice of psychiatric nurses as they provide guidelines on how care should be provided.^7^ The nursing care of those with BPD is demanding for psychiatric nurses.^8^ Therefore, supporting psychiatric nurses with frameworks or models could assist them to improve care they render to those with BPD and their significant others, and empower them. According to Cicolini and colleagues,^9^ structural and psychological empowerment from models relates to satisfying work. Empowering the psychiatric nurses is essential to increase the quality of their work leading to effective patient care and workplace satisfaction.^10,11^ This implies that better outcomes could be achieved from the use of models that provide a framework of reference in the treatment of mental health care users and their significant others.^11^

Findings from a study conducted in China^12^ assert that models provide a set of frameworks to guide nursing practice. Models thus also offer a structure that can clearly identify the requirements of nursing care. For the psychiatric nurses, models contribute to professional development. This is affirmed by Rusch and colleagues,^13^ who highlighted in their work that models promote knowledge, competence and professional attribute development effectively. Integrating models in patient care may lead to better performance of skills and enhance quality of care while boosting clinical self-efficacy, confidence, teamwork and collaboration.

Although the model for facilitation of self-empowerment for women living with BPD exists in South Africa,^14^ there is none that exists for couples living with BPD. The researcher’s previous work focussed on developing a model for psychiatric nurses to facilitate constructive intra- and interpersonal relationships for couples living with BPD.^15^ The model was developed following an observation that couples living with BPD experience intra- and interpersonal relationship challenges. The model was developed for psychiatric nurses to facilitate constructive relationship of couples in a relationship where one partner is living with BPD through a structured three-phase approach. The phases are: phase one: relationship phase, which was aimed at building a therapeutic relationship with the couple while assessing their intra- and interpersonal relationships; phase two: working phase, which focussed on guiding the couples on building positive intra- and interpersonal relationships through a process of self-awareness and personal growth, emotional regulation and the development of effective relationship skills; finally, phase three: termination phase, which was aimed at empowering the couple to manage their relationship effectively and independently while navigating the complexities of living with BPD.^15^ Based on this, the researchers noted that a model of this kind had not been implemented before by psychiatric nurses to facilitate constructive intra- and interpersonal relationships for couples living with BPD. Thus, the need to obtain the psychiatric nurses’ experiences arose following the use of the model.

## Research method and design

The study was conducted in two phases. Phase one was a workshop where the psychiatric nurses were trained on implementing the model to facilitate intra- and interpersonal relationship, and phase two used a qualitative, exploratory and descriptive research design to evaluate the model’s implementation.^16,17^

### Phase one: Workshop

A 1-day workshop was held with the psychiatric nurses as a means of training on the model. The purpose of the workshop was to facilitate comprehension of the model prior to implementation. Invitations were sent out early in September 2018 to psychiatric nurses working in units where persons diagnosed with BPD are treated, inviting them to participate in the workshop. As such, six psychiatric nurses attended and participated in the 1-day workshop at the end of September 2018. A follow-up session to provide guidance on the implementation was done a month after the implementation workshop in October 2018. During the follow-up session, challenges such as identifying couples on whom the psychiatric nurses would implement the model on were addressed. The model was implemented for 3 months from October 2018 to December 2018 by the psychiatric nurses in the units where they admitted persons diagnosed with BPD.

### Phase two: Exploratory-descriptive qualitative design

In phase two, an exploratory-descriptive qualitative design was utilised to explore and describe the psychiatric nurses’ experience of implementing the model after 3 months of implementation of the psychiatric model between January and February 2019. The use of qualitative design was aimed at gaining insights from the psychiatric nurses following implementation of the model.^18,19^ Furthermore, an exploratory-descriptive qualitative design was deemed suitable for this study as it was conducted where there is dearth of research on the topic of interest aimed at obtaining rich detailed descriptions from the psychiatric nurses who implemented the model so as to better understand their experiences.^20,21^ The six psychiatric nurses were purposefully selected to participate in this study. Sample size was determined by data saturation and data adequacy where the participants had no information and ideas to share.^14^ They met the following inclusion criteria: psychiatric nurses working in units where persons diagnosed with BPD were admitted, and able to implement the model for 3 months following the workshop. However, only four psychiatric nurses were able to implement the model and were invited for an in-depth interview following the implementation.

### Setting

The study was conducted at a specialised mental health institution in Gauteng province where the one partner with BPD receives treatment. The specialised mental health institution is located in Tshwane, South Africa. Tshwane is a city in Gauteng province, classified as a Category A municipality by the Municipal Demarcation Board in terms of Section 4 of the *Local Government: Municipal Structures Act, 1998 (Act 117 of 1998).* Tshwane caters for a diverse population as a capital city. It is further divided into seven regions, and the mental health institution where data were collected falls under Region three. The institution is classified as a specialised mental health institution. In terms of treatment of couples in a relationship where one is living with BPD, only those diagnosed with BPD are treated at the institution; however, the multidisciplinary team provides mental health care holistically to include the significant others. The mental health institution has two units with 30 bed capacity each, admitting patients diagnosed with BPD, with 5 psychiatric nurses, 3 enrolled nurses and 4 assistant nurses per shift. In addition, the mental health institution has 778 functional beds with a total of 810 nursing personnel of all categories where 350 are professional nurses, 130 are enrolled nurses and 330 are auxiliary nurses at the time of data collection.

### Data collection

Data were collected using in-depth individual face-to-face interviews which were triangulated with field notes and observations to enhance the depth and validity of the findings. In-depth interviews were conducted between January and February 2019, and were effective to gather rich and thick description of the phenomenon as experienced by the participants.^22^ All participants were interviewed individually in the hospital at a time convenient for them. Each interview lasted between 30 and 45 min (mean = 32 min) and was audio-recorded with the permission from the psychiatric nurses. All interviews were conducted in English. Prior to conducting the interviews, a pretest of the data collection instrument was conducted to assess the quality of questions, and these were refined.^17^ Thereafter, the first author conducted the interviews and posed a broad, open-ended central question that gave the psychiatric nurses an opportunity to share their experience of using the model: ‘How did the model work for you?’ Subsequent questions were guided by the psychiatric nurses’ response to the central question. Data adequacy and information power guided the sufficiency and quality of the data yielded by participants.^14,22,23^ This is the point where data are regarded to be sufficient, and the phenomenon is fully described. Of the six psychiatric nurses, only four were able to implement the model for 3 months as per the implementation guide that was provided during the workshop. As such, the four psychiatric nurses were key informants enabling the researchers to elicit rich and thick descriptions of the nurses’ experience of using and implementing the model.

### Data analysis

The audio-recorded interviews were transcribed and analysed by means of thematic analysis.^15,16^ Braun and Clark’s^24^ six steps of thematic analysis were applied as follows: (1) the authors familiarised themselves with the data by reading and re-reading through the transcribed data; (2) initial codes were generated to identify meaningful features of the data; (3) the initial codes were then organised into potential themes; (4) preliminary themes were subsequently generated through a process of refining and merging the potential themes; (5) themes were defined and named to capture the core idea of each theme; and (6) in the final step, a report was produced to present a narrative that connected the themes to the research question, thus identifying and dividing the units of meaning into themes and sub-themes. An independent coder was engaged to analyse the data independent from the researchers to minimise bias, and thereafter a consensus discussion meeting was held to verify the analysis and agree on themes. The findings of the in-depth interviews were discussed in the light of available literature in the form of literature control, as in qualitative studies, literature review is deferred until after data collection and analysis to avoid biasing the analysis and interpretation of the data.^17,20^

### Rigour

Several strategies were utilised to ensure trustworthiness of the data generated from this study. As such, the interviews were conducted until data were adequate to ensure credibility. In addition, the interviews were triangulated with observations and field notes. The interviews were audio-recorded, transcribed verbatim, and direct quotations were used to present the findings, thus ensuring dependability. Moreover, the first author bracketed own perceptions during data collection to minimise bias while ensuring credibility and dependability of the findings. In addition, the psychiatric nurses were given an opportunity to confirm interpretation of the findings for accuracy from their perspective. To ensure transferability, the researchers provided thick descriptions of methods used in the study as well as a detailed description of the participants and the study setting.

### Ethical considerations

Ethics approval to conduct the study was obtained from the University of Johannesburg Faculty of Health Sciences on 29 March 2017 (REC-01-17-2017) and the Higher Degree Committee on 10 April 2017 (REC-01-17-2017).

The psychiatric institution where the psychiatric nurses were accessed gave permission for the study to be conducted. The psychiatric nurses were approached directly by the researcher where the study information was explained, and they voluntarily signed consent form prior to participating in the study. Confidentiality and anonymity were maintained during this phase by not using identifying information that will link the participants to the data.

## Results

The demographics of the participants and the themes that emerged during data analysis are detailed in the following sub-sections.

### Description of demographics

Only six psychiatric nurses were available to participate in this study given that only two units were admitting persons diagnosed with BPD with at least five psychiatric nurses per unit (refer to description of the setting). Out of the six invited nurses, four (67%) were able to implement the model on the couples for the 3 months, while the other two were unable to complete the 3 months period of using the model. The psychiatric nurses were predominately males (75%) and had worked an average of 2.7 years with couples living with BPD. An overview of the demographic characteristics of all the participants is provided in Table 1.

**TABLE 1:** Illustration of demographics of the study participants.

Study participants	Age (years)	Gender	Educational qualification	Years of experience	Experience of working with couples living with BPD
Participant 1†	29	Male	Bachelor’s degree in Nursing	5	2 years
Participant 2	42	Female	Bachelor’s degree in Nursing BCur Hons (Advanced Psych)	12	6 months
Participant 3†	35	Male	Bachelor’s degree in Nursing	6	1 year
Participant 4	31	Male	Bachelor’s degree in Nursing BCur Hons (Advanced Psych) B Education B Admin	6	2 years
Participant 5†	44	Male	MCur Psychiatric Nursing Nursing admin	17	5 years
Participant 6†	34	Female	MCur Psychiatric Nursing	10	3 years

BPD, borderline personality disorder.

† , Represents the participants who were able to use the model for 3 months.

### Presentation of key themes

Four themes that emerged from the data are: (1) the model served as a helpful tool for empowerment; (2) the positive results yielded by the model; (3) challenges related to using the model; and (4) suggestions for expanding the model’s reach (see Table 2).^25^ Verbatim quotes are presented to support the findings as means of ensuring consistency and accuracy.^22^

**TABLE 2:** Overview of themes.

Themes	Sub-themes
1. A helpful tool for empowerment	1.1 Enabling tool for the psychiatric nurses 1.2 Supporting tool for the couples
2. Positive results yielded by the model	2.1 Transformation of the couple relationship 2.2 Improved interaction within the couple relationship
3. Challenges related to the implementation of the model	3.1 Difficulty in building trust with the couples 3.2 Difficulty engaging in the relationship phase
4. Suggestions for expanding the model’s reach	4.1 Incorporation of the model in nursing curricula 4.2 Application of the model beyond healthcare, particularly in diverse professional relationships 4.3 Application in organisational context

*Source:* Allen JG. Becoming trustworthy in treating patients with borderline personality disorder. J Pers Disord. 2023;37(5):604–619

#### Theme one: A helpful tool for empowerment

The psychiatric nurses found the model as a helpful tool to guide them to facilitate constructive intra- and interpersonal relationships with the couples living with BPD. As such, the model boosted the confidence of the psychiatric nurses enabling them to work better with the couples in relationship where one is living with BPD:

‘The model was nicely outlined at the workshop that when I used it. I am also excited for myself as I feel very empowered.’ (Participant 6, 34 years, female)

‘Model itself is very helpful and well-designed, helpful a lot because this tool was used by me and other staff members also willing to use it to also help them deal with mental.’ (Participant 1, 29 years, male)

The psychiatric nurses further acknowledged that the model empowered the couple to take control of their relationship. Therefore, serving as a tool to support the couples in a relationship where one is living with BPD:

‘It made it easy for the couple to follow through.’ (Participant 6, 34 years, female)

‘[*T*]he model or tool that I had it made things very simple because we worked along all the phases together with the couple, especially for the partner without borderline personality disorder because at the end he felt more empowered.’ (Participant 1, 29 years old)

‘Would say the model was successful in terms of implementation, simple because they (the couple) could come over and over again.’ (Participant 3, 35 years, male)

‘Exactly because they now felt they (couple) have been empowered initially there was disempowerment, but this strikes a sign of empowerment.’ (Participant 5, 44 years, male)

#### Theme two: The positive results yielded by the model

The psychiatric nurses noted positive results from the couples after implementing the model. There were noticeable changes about how the couples related to each other including learning of new skills, taking more responsibility and accountability of their actions within the relationship:

‘They (couple) learnt new skills and they were looking forward to continue with the journey of implementing changes in their relationship.’ (Participant 5, 44 years, male)

‘[*T*]hey started be accountable and be responsible for their action based on the psychoeducation that was provided to them.’ (Participant 1, 29 years, male)

‘Let me just say he was taking responsibility and accountability for actions.’ (Participant 6, 34 years, female)

The psychiatric nurses reported that the implementation of the model facilitated improvement in the couples’ interaction. This was noted in the way the couples communicated with each other during the model implementation process:

‘Their communication started improving because of the tasks that were given to them.’ (Participant 1, 29 years, male)

‘Effective in a sense that they started communicating more together as they started planning activities together.’ (Participant 3, 35 years, male)

#### Theme three: Challenges related to the implementation of the model

The psychiatric nurses experienced challenges during the initial stages of the model implementation. The challenges were mostly in the relationship phase, which included involving the partner without BPD, building trust, explaining BPD as well as starting the process of introspection:

‘During that phase the problem, one partner is not having the condition and the other is having the condition (referring to one partner in the couple relationship living with BPD and the other not). So now having to align both of them so that they can be in the same boat, it was little bit of a hiccup.’ (Participant 5, 44 years, male)

‘It’s difficult for someone you do not know to share your problems with them without knowing them, trying to build trust and trying to understand and respect them and their problems.’ (Participant 1, 29 years, male)

‘It was difficult to get their partners to come in.’ (Participant 3, 35 years, male)

‘To ensure proper therapeutic relationship.’ (Participant 6, 34 years, female)

Although it was difficult to initiate the model implementation, the psychiatric nurses experienced the couples to have engaged well in the working phase and they realised the importance of preparing the couple for termination phase during the process of model implementation. The model helped them to direct the process of implementation:

‘It helped to direct the process as it is not easy to be a third person in a couple’s space trying to understand how.’ (Participant 5, 44 years, male)

‘After building initial trust with each of them as individuals and them I spoke to them as a couple.’ (Participant 3, 35 years, male)

#### Theme four: Suggestions for expanding the model’s reach

After noting the transformation on the couple relationship, the psychiatric nurses made suggestions on the applicability of the model. The psychiatric nurses suggested that the model be applied to health care education. The psychiatric nurses experienced the model as beneficial and therefore suggested that it be incorporated in health care education, particularly for the training of nursing students in psychiatric nursing. The psychiatric nurses thought the model can assist nursing students to advance their knowledge and skills in facilitating the relationships of couples in a relationship where one person is diagnosed with BPD:

‘It should be incorporated into the curriculum for the students because the students in real practice, these are the situations they come across.’ (Participant 3, 35 years, male)

‘To the community and also universities should also use this model’. (Participant 5, 44 years, male)

‘It (the model) must be ensured that the students are using it. They are applying it whenever they are on practicals.’ (Participant 6, 34 years, female)

‘They (couples) should be taught with the model; the model should be incorporated, and it must be ensured that the students are using it.’ (Participant 1, 29 years, male)

The psychiatric nurses further suggested that the model be applied in other relationships such as relationships of the couples who are dealing with other chronic conditions as well as professional relationships:

‘Any day-to-day relationship can benefit from this model because most of the relationships are having challenges and employees can be taught about this model so that it can be implemented at broader spaces.’ (Participant 5, 44 years, male)

‘I think it is very useful and not just with couples in a relationship where one is living with borderline personality disorder but other relationships that are affected by presence of chronic illness.’ (Participant 6, 34 years, female)

Not only did the psychiatric nurses suggest application of the model to other relationships but also in an organisational context where there are challenges with interpersonal relationships:

‘The workers should be taught about the model.’ (Participant 3, 35 years, male)

‘Other institutions there are employee wellness programs, maybe you can go there and sit people who are directing those programmes and sell this idea to them.’ (Participant 1, 29 years, male)

‘Employees can be taught about this model so that it can be implemented at broader spaces.’ (Participant 5, 44 years, male)

## Discussion

The findings of this study provide valuable insights into the psychiatric nurses’ experiences of implementing a model developed to facilitate constructive intra- and interpersonal relationships of couples in a relationship where one partner is living with BPD. The psychiatric nurses experienced the model as a helpful tool to facilitate intra- and interpersonal relationships of couples navigating the challenges associated with BPD, at the same time guiding them while providing care. The findings of this study align with those of Han, Zhu and Chen highlighting that models provide a framework for guiding nursing practice.^12^ The participants of this study reported the model as a helpful tool for empowerment, both for themselves and for the couples they worked with. Psychological empowerment is an individual’s perceived sense of meaning and purpose, competence and impact on one’s role.^26^ The model empowered the psychiatric nurses and provided a structured approach that boosted nurses’ confidence in working with BPD-affected relationships. This finding is consistent with previous research that highlighted the importance of evidence-based tools in enhancing healthcare providers’ self-efficacy.^27^ Healthcare providers’ self-efficacy appears to increase with training and support, making this model relevant to provide efficacy for psychiatric nurses to facilitate constructive intra- and interpersonal relationships for couples in a relationship where one partner is diagnosed with BPD by providing a guiding framework of care for such couples.^10,13^ Similarly, a study by Farrel and colleagues^28^ found that implementing evidence-based guidelines increased clinicians’ self-efficacy.

Moreover, the model’s ability to empower couples to take control of their relationship suggests its potential for promoting patient autonomy and engagement – key factors in successful mental health interventions. The psychiatric nurses revealed positive results yielded by the model, including noticeable transformations in couple relationships and improved interactions. In addition, the couples learned new skills, took more responsibility for their actions and improved their communication. These outcomes are particularly significant given the interpersonal difficulties often associated with BPD.^29,30^ The model’s success in facilitating these changes suggests its feasibility as an effective intervention for addressing the relationship challenges in BPD because it provides support to both the psychiatric nurses and the couples. Literature suggests that such interventions could positively impact on patient care while providing psychological support as they receive care.^31^

An interesting finding was the psychiatric nurses’ experience that the model could enhance their own professional practice. Many reported that engaging with the model increased their understanding of the complex dynamics within the relationships of those with BPD and provided them with new tools for intervention. This suggests that implementing such a model could have secondary benefits in terms of professional development and job satisfaction among psychiatric nurses who find it challenging to provide care to persons with BPD. As such, the acceptability of the model among psychiatric nurses was generally positive, with many expressing enthusiasm about its potential to fill a gap in current treatment. The acceptability of the model implemented by the psychiatric nurses in this study aligns with the growing recognition of the importance of systemic interventions in BPD treatment such as the model implemented for women diagnosed with BPD.^32^

Although the psychiatric nurses seem to have an opinion that the model is effective to facilitate constructive intra- and interpersonal relationships of couples where one is living with BPD, they experienced challenges such as building trust, particularly with the one partner who is not living with BPD at the initial stages. These challenges highlight the complexities of working with persons in a relationship where one is diagnosed with BPD and the importance of the relationship-building phase in therapeutic interventions. In their study, Griffith and Johnson described that the initial step in developing a trusting relationship is demonstrating respect as it lays the groundwork for trust.^33^ Being genuine and honest contributes to the development of a trusting relationship making these attributes important to build trust. As such, cultivating a trusting relationship with a person diagnosed with BPD is ideal for therapeutic interactions.^25^ In addition, a study conducted on women diagnosed with BPD indicated the challenges experienced in therapeutic relationships requiring a degree of flexibility to foster security in the therapeutic relationships.^34^ Therefore, future refinements of the model might need to focus on strategies to overcome these initial hurdles, possibly by incorporating elements of communication techniques from established trust-building techniques in couples in therapy.^35^

The psychiatric nurses suggested broader application of the model – incorporating the model into nursing curricula, applying it to other types of relationships affected by chronic conditions and even extending its use to organisational contexts. This enthusiasm for wider application indicates the acceptability and value of the model beyond its original scope.

### Strengths and limitations

While the study provides rich insights, because of its qualitative nature and small sample size, generalisability is limited. The study’s findings are based on a single urban mental health institution. While this context is essential, the findings were limited. Future research should apply this model to other settings, such as rural contexts. In addition, with regard to the smaller sample size, the findings should be interpreted with caution. To mitigate this, data adequacy was used which is when the participants share no new information and ideas. Finally, the study focusses on psychiatric nurses’ perspectives alone. Future research should incorporate the views of individuals with BPD, their partners and other mental health professionals to provide a more comprehensive understanding of the model’s feasibility and acceptability.

### Recommendations

The insights gained from this study can be used to support psychiatric nurses and other mental health professionals who work with couples living with BPD and other mental health conditions. Based on the findings of this study, recommendations are made for clinical practice. Therefore, the model could be used in broader spaces where the psychiatric nurse facilitates the mental health of couples living with any chronic illness.

The model could benefit other relationships as well as the society. This model could be used in the training of student nurses in psychiatric nursing to equip them with knowledge and skills to manage such couples including training of other mental health professionals. The authors further recommend that the model be implemented in other contexts. Research where both spouses in the relationship are living with BPD is warranted to help understand their experiences of the relationship and possibly another model similar to the one under study could be developed to improve the relationship of both spouses.

## Conclusion

This study revealed that the model was feasible and acceptable to achieve constructive intra- and interpersonal relationships of couples in a relationship where one partner is living with BPD. Based on this, the use of model such as the one implemented in this study provides a framework of reference for psychiatric nurse to carry out their duties with ease. While psychiatric nurses generally experienced the model as feasible and acceptable, several areas for refinement and further investigation were identified necessitating future research. These findings provide a foundation for future research and development of interventions aimed at improving outcomes for couples whose relationship is affected by BPD.
